# Second-Generation Antipsychotics and Dysregulation of Glucose Metabolism: Beyond Weight Gain

**DOI:** 10.3390/cells8111336

**Published:** 2019-10-29

**Authors:** Diana Grajales, Vitor Ferreira, Ángela M. Valverde

**Affiliations:** 1 Instituto de Investigaciones Biomédicas Alberto Sols (CSIC-UAM), 28029 Madrid, Spain; dgrajales@iib.uam.es (D.G.); vdasilva@iib.uam.es (V.F.); 2 CIBER de Diabetes y Enfermedades Metabólicas Asociadas (CIBERDEM), ISCIII, 28029 Madrid, Spain

**Keywords:** antipsychotics, second-generation antipsychotics, glucose dysregulation, insulin resistance, insulin secretion, metabolic side-effects, schizophrenia, type 2 diabetes

## Abstract

Second-generation antipsychotics (SGAs) are the cornerstone of treatment for schizophrenia because of their high clinical efficacy. However, SGA treatment is associated with severe metabolic alterations and body weight gain, which can increase the risk of type 2 diabetes and cardiovascular disease, and greatly accelerate mortality. Several underlying mechanisms have been proposed for antipsychotic-induced weight gain (AIWG), but some studies suggest that metabolic changes in insulin-sensitive tissues can be triggered before the onset of AIWG. In this review, we give an outlook on current research about the metabolic disturbances provoked by SGAs, with a particular focus on whole-body glucose homeostasis disturbances induced independently of AIWG, lipid dysregulation or adipose tissue disturbances. Specifically, we discuss the mechanistic insights gleamed from cellular and preclinical animal studies that have reported on the impact of SGAs on insulin signaling, endogenous glucose production, glucose uptake and insulin secretion in the liver, skeletal muscle and the endocrine pancreas. Finally, we discuss some of the genetic and epigenetic changes that might explain the different susceptibilities of SGA-treated patients to the metabolic side-effects of antipsychotics.

## 1. Introduction

### 1.1. Antipsychotic Treatment for Schizophrenia and Other Mental Illnesses: First- Versus Second-Generation Antipsychotics

Antipsychotic drugs (APDs) are the cornerstone of treatment for schizophrenia and other mental diseases, including bipolar disorders, dementia, autism-related irritability and severe mental illness [1,2,3]. Since the release of the first antipsychotic—chlorpromazine—in 1952, which was originally synthesized as an anesthetic adjunct, pharmacotherapy has been the mainstay treatment for psychotic disorders [4]. The clinical success of chlorpromazine for treating schizophrenia stimulated the widespread synthesis of chlorpromazine-related compounds, leading to the later development of the so-called first-generation antipsychotics (FGAs) (also known as typical antipsychotics) such as fluphenazine and haloperidol, whose common features were the tricyclic chemical structure of chlorpromazine and their ability to induce catalepsy in rats [5]. However, many patients treated with FGAs had a suboptimal response and developed extrapyramidal side-effects (EPS) and severe movement disorders, such as parkisonism, dyskinesia and akathisia. These serious symptoms prompted the development of new compounds, known as second-generation (atypical) antipsychotics (SGAs), which did not produce significant EPS and preserved the efficacy for treating refractory schizophrenia [6,7]. The first of these SGAs was clozapine, which was introduced in the mid-1970s, and was followed by risperidone, olanzapine, ziprasidone, quetiapine, amisulpride, sertindole, lurasidone, paliperidone, iloperidone, asenapine and, more recently, aripiprazole [8]. The APDs have in common their antagonism of the D2 dopamine receptor (D2R) with the exception of aripiprazole, which acts as a partial agonist [9]. It has been estimated that APDs show optimal efficacy when their D2R occupancy is 60–70%, and D2R occupancy levels higher than 80% frequently induce EPS. Because SGAs maintain a threshold of 65–80% D2R occupancy, their use minimizes the risk of EPS while achieving optimal antipsychotic efficacy [10,11,12,13]. The elevated D2R occupancy of some FGAs (such as haloperidol) was recently reported to result from their fast association rate and high potential of rebinding, whereas the slower association rate and lower rebinding potential of SGAs reduce D2R occupancy [14].

In contrast to the exclusively high affinity binding of FGAs to D2R, SGAs exert their action by engaging with a broader range of receptors, including dopamine (D1R, D2R and D4R), serotonin (5-HT_1_A, 5-HT_2_A, 5-HT_2_C, 5-HT_6_, 5-HT_7_), histamine (H1R) and muscarinic (M3R) receptors [15,16]. In addition to the reduction in D2R occupancy, the lower liability of EPS with SGAs can be explained by their antagonism or inverse agonism of the serotonin 5-HT_2_A receptor, which can increase dopaminergic transmission in the nigrostriatal pathway (impeding the development of motor disorders), and agonism of 5-HT_1_A in the mesocortical pathway, which facilitates dopamine release in the prefrontal cortex and hippocampus [17]. Overall, the switch from exclusively D2R antagonism of FGAs to redesigned SGAs, which have a more diverse receptor binding profile, has improved the negative symptoms (including EPS) and the tolerability in patients and, hence, SGAs are extensively prescribed as the first-line of treatment for schizophrenia.

### 1.2. Are the Metabolic-Side Effects in Patients with Severe Mental Illness Due to Schizophrenia, SGAs or Other Factors?

It has been estimated that the lifespan of patients with severe mental illness is reduced by 10–20 years compared with the healthy population [18], with cardiovascular disease being the leading cause of death in people with schizophrenia [19,20,21]. In fact, patients with schizophrenia are at three-fold higher risk of obesity and two- to four-fold higher risk of type 2 diabetes mellitus (T2D), which are common causes of cardiovascular disease [22]. Several factors are involved in the increased risk of cardiovascular disease in this population, including higher rates of smoking [23], unhealthy diet and reduced physical exercise [24,25], low-income [26] and the antipsychotic treatment itself, particularly SGAs [27]. Indeed, it is now recognized that despite their clinical efficacy in treating psychiatric disorders and reducing motor side-effects over FGAs, SGAs are associated with severe metabolic alterations such as weight gain, hyperphagia, hyperglycemia, insulin resistance, dyslipidemia, and T2D development—all common features of the metabolic syndrome (MetS) and major risk factors for cardiovascular disease [28].

The possibility that severe mental illness, and especially schizophrenia, is independently associated with the development of MetS and cardiovascular disease, has been investigated in several studies, with results showing that first-episode, drug-naïve patients with schizophrenia have increased plasma insulin levels [29,30], elevated fasting glucose [31], impaired glucose tolerance [32], and changes in lipid metabolism [28]. Given these findings, the possibility of a genetic predisposition to both T2D/weight gain and schizophrenia has been examined; however, results so far have been inconclusive mainly because of the polygenic contribution to both diseases, which challenges the identification of allelic risk variants. Nevertheless, some candidate genes associated with T2D and schizophrenia have been proposed, including phosphatidylinositol 3-kinase regulatory subunit alpha (*PIK31*), an upstream activator of protein kinase B (AKT) signaling; calcium-dependent mediators such as protein kinase C alpha (*PRKCA*) and protein kinase, DNA-activated, catalytic subunit (*PRKAC*); non-receptor type 11 protein tyrosine phosphatase (*PTPN11*) [33], which is involved in adipokine signaling; protein tyrosine phosphatase receptor type D (*PTPRD*) [34]; and low-density lipoprotein receptor-related protein (*LRP4*) [35]. Yet, other studies have not found a clear pathogenetic association between the two diseases [36,37].

There is much stronger evidence directly linking SGA medication to increased risk of T2D in patients with schizophrenia. In fact, is well known that the pediatric population is especially susceptible to the metabolic side-effects of SGAs [38,39]. In a study in young patients [38], first-time use of olanzapine, risperidone, aripiprazole or quetiapine induced the transition from normal weight to overweight/obese status in 10–36% of individuals after only 12 weeks of treatment, with olanzapine inducing the strongest effects on body weight gain. Similarly, the European first-episode schizophrenia trial study (EUFEST) reported that whereas the prevalence of MetS was similar between young antipsychotic-naïve patients with schizophrenia and the normal population, a worsening of glucose levels leading to hyperglycemia was noted in those patients treated with amisulpride, olanzapine or ziprasidone for 52 weeks [27]. This is in line with the evidence of a recent large-scale systematic review and meta-analysis [25] that found a prevalence of only 2.9% of T2D in antipsychotic-naïve patients and 11.3% in patients with severe mental illness medicated with SGAs, with the highest prevalence associated with clozapine and olanzapine. A recent nationwide Danish study also reported a significant relationship between exposure to SGAs and diabetic ketoacidosis, caused by insulin deficiency [40]. In another Danish cohort, olanzapine and aripiprazole were shown to increase the rate of T2D in patients with schizophrenia by almost two-fold, whereas clozapine increased the rate four-fold [41]. Of note, the association between aripiprazole (which acts as a partial agonist of D2R) and metabolic-side effects remains unclear, as some studies have found a lower risk for T2D in patients treated with this drug [25,42]. Moreover, it has been shown that genetic polymorphisms can influence the pharmacokinetics of SGAs. For example, polymorphisms in P-glycoprotein, a drug efflux pump, impact the pharmacokinetics of SGAs and, consequently, how patients respond to pharmacotherapy [43].

While FGAs were not initially linked to metabolic side-effects, some studies indicate that both FGAs and SGAs increase the risk for T2D. In a large Danish cohort study (7139 subjects) first-episode, drug-naïve patients with schizophrenia receiving the FGAs chlorpromazine, levomepromazine or chlorprothixene had an elevated risk for T2D, although the highest risk was found in patients prescribed the SGAs olanzapine or clozapine [42]. In a similar line, a significant increase in body-mass index was reported in South African first-episode, drug-naïve patients with schizophrenia treated for 12 months with long-acting injectable flupenthixol, and this was accompanied by increased triglyceride and decreased high-density lipoprotein levels [44]. Nevertheless, as mentioned above, the majority of studies comparing FGAs *versus* SGAs report that metabolic abnormalities are more common in patients prescribed the latter [45,46]. Furthermore, it is generally accepted that the pediatric and female populations are more susceptible to the metabolic side-effects of SGAs [47].

**Figure 1:**
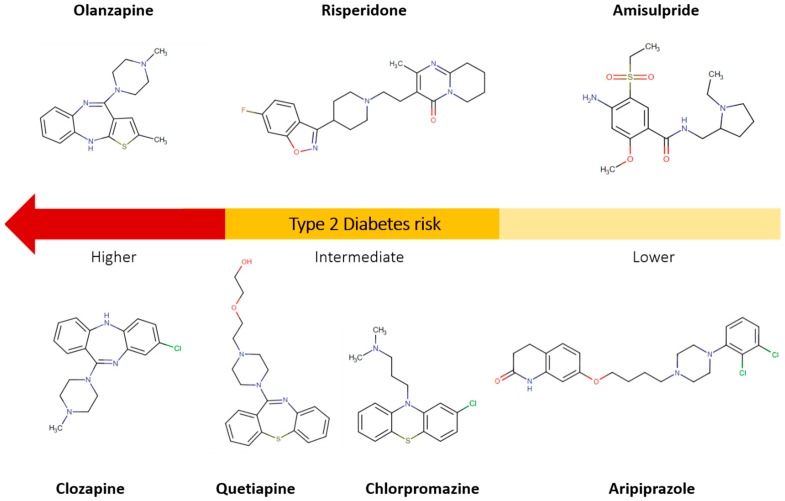
Scheme of antipsychotic drugs (APDS) commonly associated with diabetogenic properties. First-generation antipsychotic (FGA) = chlorpromazine; Second-generation antipsychotics (SGAs) = olanzapine, clozapine, quetiapine, risperidone, amisulpride and aripiprazole.

Unraveling the association between severe mental illness, SGAs and T2D is a challenging endeavor, as most epidemiological studies have limitations. The inability to adjust for confounding factors such as age, sex, specific treatment (posology and duration), previous treatment/s or switch of medication, onset and severity of severe mental illness, variation in the sample size, lifestyle, and socio-economic factors, can all have an impact on outcomes. Moreover, each SGA can have a different metabolic risk profile independently of its class and, in this regard, it is perhaps more valuable to assess the individual risk of each drug rather than focusing on the collective metabolic side-effects of one class or the other. Overall, even if a schizophrenia-related locus can confer an increased risk for MetS, this susceptibility is further bolstered by APD medication, primarily SGAs, with variable and complex metabolic side-effects and diabetogenic properties (Figure 1).

### 1.3. Elucidation of SGAs Effects on Glucose Tolerance, Insulin Resistance and Body Weight Gain: Acute Studies with Healthy Volunteers

While accumulating evidence has linked SGAs therapy to metabolic disturbances, understanding the mechanistic bases of these side-effects remains challenging as the bulk of research is conducted in patients under chronic treatment. Accordingly, short-term administration of SGAs in healthy volunteers is commonly performed to examine the primary metabolic side-effects independently of the secondary effects triggered by T2D and obesity, as well as other disturbances.

Since olanzapine and clozapine appear to induce the most severe metabolic abnormalities, they are, understandably, the focus of the bulk of studies in healthy volunteers. In this regard, acute effects of these drugs on glucose tolerance (≤14 days) have been evaluated in several studies. For example, in an open-label study, healthy volunteers with no family history of inherited T2D, fasting glucose levels ≤100 mg/dL and normal body-mass index (18–25 kg/m^2^) were randomized to receive olanzapine (10 mg/d) or ziprasidone (80 mg/d) for 10 days, and were then subjected to hyperinsulinemic-euglycemic clamp (HEC) analysis, which measures insulin action in vivo. Olanzapine was found to reduce glucose uptake and increase plasma insulin levels compared with baseline levels, whereas ziprasidone failed to cause acute changes in glucose tolerance [48]. In another study in healthy subjects without psychiatric history and with normal body-mass index, olanzapine or aripiprazole (10 mg/d) were administered for 9 days and insulin sensitivity was evaluated after conducting a meal challenge before and after HEC analysis [49]. Olanzapine induced postprandial hyperinsulinemia in 9 out of 10 subjects, accompanied by a small elevation in glucagon levels and, surprisingly, an increase in GLP-1 levels, which are normally reduced in T2D. Similar to olanzapine, aripiprazole decreased the C-peptide/insulin ratio, considered as an index of hepatic insulin clearance and insulin resistance. No changes in body weight were reported in individuals receiving either of the SGAs, suggesting that metabolic changes in insulin-sensitive tissues are triggered before body weight gain. The rapidly induced metabolic effects of olanzapine have also been reported with regard to lipid metabolism, as only a 3-day treatment (10 mg/d) in healthy volunteers increased circulating leptin and triglycerides levels, typically elevated in T2D [50]. However, individuals have a different susceptibility to SGA-induced metabolic side-effects. For instance, in a study of healthy male volunteers, mild treatment with olanzapine over two weeks (5 mg/d for the first 7 days and 10 mg/d for the next 7 days) led to weight gain in only two-thirds of volunteers. Nevertheless, all volunteers showed a significant increase in circulating insulin, triglycerides and leptin, but only the weight-gain group showed changes in glucose, low-density lipoprotein and total cholesterol levels [51]. A meta-analysis of healthy volunteers’ studies showed that weight gain is typically found in treatments lasting longer than 14 days, clearly associating body weight gain with the length of the treatment, whereas insulin sensitivity is more closely associated with the SGA used [52]. In this regard, a cross-sectional analysis of more than 3000 articles published on antipsychotic-induced weight gain (AIWG) in first-episode, drug-naïve patients with schizophrenia found an average increase of 5.3 kg in weight and 1.86 kg/m^2^ in body-mass index in SGA-treated patients compared with patients receiving placebo in treatments longer than 12 weeks; the highest weight gain was found with olanzapine and clozapine, followed by quetiapine and risperidone. This meta-analysis also found that aripiprazole, often associated with fewer metabolic side-effects, led to weight gain after short-term periods of treatment [53].

Despite this review focuses on SGAs-mediated glucose dysregulation independently of body weight gain, it is noteworthy to mention that both AIWG and dyslipidemia are major metabolic side-effects [28,50,51]. AIWG results from the imbalance between energy intake and energy expenditure and the variety of receptors that SGAs target suggest that AIWG is a multifactorial phenomenon. While the mechanisms behind reduction in energy expenditure are not fully understood [54], meta-analysis studies have correlated the ability of SGAs to target neurotransmitter receptors in the central nervous system (CNS) with increased food intake. The more relevant include blockade of hypothalamic H1R [55], different polymorphisms in serotonin receptor 5-HT_2_C [56,57], that interfere with satiety and appetite centers, and blockade of D2R receptors [58] which might hamper the normal functioning of reward-related behaviors, also explaining the weight gain susceptibility observed in SGAs-treated patients. Dyslipidemia, on the other hand, is commonly manifested as an increase in total triglyceride and decrease of high-density lipoprotein levels. It is well known that the liver converts the excess of exogenous glucose into triglycerides. Triglycerides are then packaged into low-density lipoproteins and transported to the adipose tissue for long-term energy storage [59]. Therefore, as we will discuss in the following sections, the ability of SGAs to increase blood glucose levels and induce insulin resistance can also misbalance triglyceride metabolism and cause dyslipidemia. On the other hand, increased cholesterol levels could be due to a direct effect of SGAs in lipid metabolism independently of changes in glucose metabolism, as these drugs up-regulate sterol regulatory element binding protein-2 (SREBP-2) [60,61], the master regulator of cholesterol biosynthesis.

Overall, studies in healthy volunteers link SGAs to body weight gain, but also to impairment in glucose metabolism independently of AIWG, the main subject of this review. However, the short duration of the clinical trials might underpower the link between SGAs and T2D, since T2D development occurs over many years. Despite this limitation, studies with healthy volunteers have contributed significantly to address the sub-acute metabolic effects of SGAs and their diabetogenic properties, which might predict the outcome of chronic long-term treatment with these drugs in patients.

## 2. New Insights into the Molecular Mechanisms by which SGAs Disrupt Whole-Body Glucose Homeostasis: From Cellular and Animal Models to Humans

It was initially believed that glucose dysregulation linked to SGAs results from AIWG, but it is now accepted that glucose metabolism is dysregulated even in the absence of weight gain. Therefore, this review is based on the dysregulation of glucose homeostasis by SGAs independently of body weight gain and changes in lipid metabolism/adipose tissue function, focusing on the molecular alterations triggered by SGAs in liver, skeletal muscle and endocrine pancreas. In this regard, as we will discuss below, cellular and preclinical animal models have provided valuable knowledge about the main targets and molecular pathways disrupted by SGAs (for instance, the intracellular insulin signaling pathways) that can predict long-term metabolic outcomes.

### 2.1. Cellular Studies Addressing Dysregulation of Glucose Homeostasis by SGAs

Mechanistic insights into the cell-autonomous signaling pathways targeted by SGAs have been gained using in vitro studies. An important caveat in translating these results to a pathophysiological in vivo context, however, is the difference between the therapeutic plasma levels found in patients and the doses used in vitro. For instance, the therapeutic plasma levels of olanzapine and clozapine are approximately 0.3 to 3 μM respectively [62], whereas the concentrations used in vitro normally range between 1 and 100 μM. That being said, it has been reported that the concentration of SGAs in rodent tissues could be 20–100-fold higher than those in plasma [63,64].

#### 2.1.1. SGA-Mediated Molecular Alterations in Glucose Metabolism in Hepatocytes: In Vitro Studies

Hepatocytes play a critical role in glucose homeostasis by regulating glycolysis, gluconeogenesis, glucose uptake and glycogen synthesis [65]. However, very little is currently known about the impact of SGAs on the molecular machinery that governs glucose metabolism in hepatic cells.

Hepatic glycogen and triglycerides are the two long-term sources of energy storage in the body. Glycogen accumulates in the liver in response to insulin and is hydrolyzed to glucose in response to glucagon, ensuring the balance in overall glucose homeostasis. Hepatic glycogen synthesis is regulated by glucokinase and glycogen phosphorylase. Activation of glucokinase increases the flux of glucose towards glycolysis and glycogen synthesis, whereas inactivation of glycogen phosphorylase stimulates glycogen storage. Serotonin signaling is known to exert both stimulatory (via 5-HT_1_A/5-HT_2_A receptors) and inhibitory (via 5-HT_2_B receptors) effects on hepatic glycogen synthesis, with the stimulatory action dominating. This was elegantly elucidated by Tudhope and collaborators [66], who showed that the double 5-HT_1_/5-HT_2_ agonist α-methyl-5HT, which mimics the stimulatory but not the inhibitory effects of 5-HT, increased glycogen synthesis in rat hepatocytes after 2 h incubation at 100 μM concentration. This effect was blunted in hepatocytes co-treated with α-methyl-5HT and olanzapine (10–100 μM), which binds 5-HT receptors. In an earlier study, the same group reported that α-methyl-5HT stimulates glycogen synthesis by inactivating glycogen phosphorylase, and showed that clozapine and olanzapine counteracted this inactivation [67]. Therefore, inhibition of glycogen synthesis in hepatocytes might be a mechanism underlying the hyperglycemia associated with both SGAs.

In addition to disrupting glucose homeostasis in hepatocytes, SGAs are also linked to hepatoxicity, but only at doses far exceeding therapeutic levels. In studies using the insulin-sensitive hepatic cell line FL-83Bm, exposure to 10–50 μM clozapine over 24 h increased mitochondrial membrane depolarization in a dose-dependent manner [68]. Supporting this finding, isolated rat hepatocytes were subjected to accelerated cytotoxicity mechanism screening, a technique used to assess the cytotoxicity of high doses of drugs during short-time periods and that apparently correlates with cytotoxicity of lower doses and longer periods, which revealed that both risperidone (400 μM) [69] and olanzapine (6 mM) [70] induce the production of reactive oxygen species (ROS), leading to mitochondrial dysfunction.

#### 2.1.2. Alterations of the Insulin Signaling Pathways and Glucose Transport in Skeletal Muscle Cellular Models

Studies in skeletal muscle cells have revealed SGA-induced impairments in glycogen content, insulin signaling, and glucose transport. Similar to hepatic cells, rat L6 skeletal muscle cells treated with olanzapine (100 μM) had diminished glycogen content concomitant with phosphorylation of glycogen synthase at Ser^641^. By contrast, amisulpride, a SGA not associated with T2D induction, failed to elicit this effect [71]. In the same study, olanzapine treatment (10 and 100 μM for 72 h) blocked insulin-stimulated insulin receptor substrate-1 (IRS-1) phosphorylation and decreased AKT and glycogen synthase kinase-3 (GSK-3α/β) phosphorylation, reflecting an impairment of the insulin signaling cascade. Likewise, clozapine (1.5 μM, 24 h) reduced insulin-induced IRS-1 tyrosine phosphorylation and AKT serine phosphorylation at Ser^473^ and inhibited glucose uptake [72]. In a study using mouse C2C12 myoblasts, olanzapine (20 μM, 2 h) failed to modulate AKT activity, both in the absence and the presence of insulin [73]; however, another study showed that it increased glucose uptake via the activation of adenosine 5-monophosphate-activated protein kinase (AMPK) [74], which is in contrast to the reported inhibitory actions of this antipsychotic on glucose uptake in C2C12 [75] and L6 muscle cells [72,76]. In a more recent study, quetiapine (10–100 μM, 1 h) treatment decreased insulin-stimulated glucose uptake in C2C12 myotubes, an effect that could be reverted by calcitriol (0.1–10 nM), highlighting the positive effect of vitamin D in alleviating SGA-induced metabolic side-effects [77]. Likewise, ex vivo experiments in rat soleous skeletal muscle showed no effects of haloperidol, olanzapine or clozapine (1–10 μM, 30 min) on glucose uptake [78]. It is relevant to mention that the impact of SGAs on the translocation of the insulin-sensitive glucose transporter (GLUT) 4 to the plasma membrane has not been studied in depth, although it was reported that olanzapine (20 μM, 2 h) stimulated glucose transport in C2C12 cells by increasing the abundance of GLUT4 at the plasma membrane, in a similar manner to insulin [73]. However, a recent study has shown that 5-HT induces GLUT4 translocation to the plasma membrane via serotonylation of Rab4, a small GTPase activated by the insulin-signaling pathway, suggesting that disruptors of 5-HT signaling, such as SGAs, may interfere with GLUT4 translocation and glucose transport in muscle cells [79]. This effect could be achieved by a direct interaction with GLUT4 as olanzapine can bind the *Staphylococcus epidermidis* glucose/H(+) symporter (GlcPSE) through a residue also conserved in this glucose transporter [80].

Overall, the limited number of in vitro studies in skeletal muscle cells have linked SGAs with insulin signaling impairment, mainly by derangement of AKT phosphorylation and interference with glucose uptake and transport, although the effects may be different depending on the doses and incubation times used.

#### 2.1.3. Impact of SGAs on Insulin Secretion and β-Cell Function: Molecular Targets in Cultured β-Cells and Pancreatic Islets

Insulin secretion is a tightly controlled process that is regulated by a number of factors including dopaminergic, serotonergic and cholinergic signaling pathways. Pancreatic β-cells express a variety of dopaminergic receptors (D1R–D2R) and can synthesize, metabolize and store dopamine inside their vesicles [81,82,83]. In response to high glucose, dopamine is co-released with insulin into the extracellular space where it exerts a paracrine action on the adjacent β-cells by binding to D2R and partially suppressing insulin secretion via decreasing cell membrane depolarization and calcium influx [84,85]. Moreover, β-cells express different serotonergic receptors and synthesize, store and release 5-HT in response to glucose stimulation, although extracellular 5-HT may act as a stimulator or inhibitor of insulin secretion depending on the expression of the different 5-HT receptor subtypes [86,87]. In addition, the signaling pathways mediated by M3R in the pancreatic islets are also tightly linked to insulin secretion and β-cell function [88,89]. Despite these numerous investigations, the impact of SGAs on insulin secretion remains unclear and seems to depend on the dose and treatment time, as well as on the binding to specific receptor subtypes. For example, static incubation of cultured human islets with sulpiride or haloperidol (500 nM), clozapine or olanzapine (0.5 and 5 μM, respectively) and 15 mM glucose for 1 h increased glucose-stimulated insulin secretion (GSIS), with D2R antagonism as the proposed mechanism [85]. In a similar study, the effect of chlorpromazine, haloperidol, perphenazine, zuclopenthixol, clozapine, olanzapine and risperidone (all used at 1 μM for 4 h) on basal insulin secretion and GSIS was investigated in rat pancreatic islets. Whereas clozapine increased basal insulin release, haloperidol inhibited GSIS, and the other drugs had no significant effects. The study authors proposed that the stimulatory effect of clozapine on basal insulin release might explain its ability to increase appetite and induce weight gain [90]. Interestingly, the same group reported an increase in basal insulin release in rat pancreatic islets and INS-1 insulinoma cells with both olanzapine and clozapine (1 μM, 4 h), and found a trend towards an increase in GSIS [91]. The same study reported an inhibition of GSIS with zuclopenthixol, a FGA. In line with these findings, a later study found that clozapine (1 or 10 μM) increased GSIS in isolated rat islets challenged with 10 mM glucose (30 min) and, surprisingly, concomitantly suppressed the inhibitory action of glucose on glucagon release from pancreatic α-cells [92]. Mechanistically, other authors found that the increase in basal insulin release in INS-1 cells at low glucose concentration in the presence of 25 μM clozapine for 48 h upregulated the transcription factor *Foxa1* and the mRNA levels of its downstream target the mitochondrial citrate carrier (*Slc25A1* or CIC). Silencing of *Foxa1* expression completely abolished basal insulin secretion in INS-1 cells treated with clozapine. By contrast, the FGA haloperidol (25 μM for 24 h) failed to modify *Foxa1* levels and had no effect on insulin secretion [93]. The authors hypothesize that activation of *Foxa1* by clozapine triggers exocytosis of insulin granules, but the molecular mechanism underlying such an effect remains to be elucidated. Overall, these in vitro studies suggest that the increase in basal insulin release and GSIS might explain the hyperinsulinemia associated with chronic treatment with SGAs in patients [48,49,94].

Disruption of M3R action on glucose-dependent parasympathetic regulation of insulin secretion by SGAs has also been postulated. Johnson and collaborators [95] reported that olanzapine and clozapine, potent M3R antagonists, suppress cholinergic-stimulated insulin secretion by directly blocking these receptors in β-cells. In their study, carbachol (10 μM), a cholinergic agonist, stimulated insulin secretion in rat islets perfused with 7 mM (low) glucose during 30 min, but co-treatment with 10–100 nM clozapine or olanzapine abolished carbachol-stimulated insulin secretion. Ziprasidone, risperidone and haloperidol, which lack affinity for M3R, did not change carbachol-stimulated insulin secretion. Of note, olanzapine, clozapine or ziprasidone alone, used at 10 μM, did not affect insulin secretion in rat islets in 8 mM glucose compared with the vehicle condition, although the effect of high glucose concentration (16.7 mM) was not reported in this study. These results were replicated by Sasaki and co-workers [96], who also reported inhibition of carbachol-stimulated insulin secretion in rat islets incubated with clozapine (1–5 μM) and carbachol (10 μM) in 7 mM glucose medium for 1 h. These researchers also explored the long-term effect of clozapine at high glucose concentration, reporting that clozapine (1 μM) inhibited GSIS by 53% and reduced intracellular Ca^2+^ and ATP levels in rat islets, an effect observed after 7 days of culture. Likewise, clozapine (1 μM) inhibited insulin secretion in rat islets stimulated by depolarization with K^+^ and diazoxide (250 μM) after incubation for 7 days, and also inhibited insulin secretion stimulated by protein kinase C activation, but only when it was used at 5 μM concentration. Therefore, it seems that clozapine suppresses ATP production in β-cells by reducing the closure of ATP-sensitive K^+^ channels and decreasing the depolarization of the plasma membrane, which in turn leads to a decrease in Ca^2+^ influx, the driver of exocytosis of insulin granules. An acute inhibitory effect of clozapine (5 μM) in isolated rat islets was reported by Best et al., following 60 min incubation [97]. Also, β-cell function was impaired by olanzapine and risperidone (100 μM) in the hamster β-cell HIT-T15 line, with both drugs inducing endoplasmic reticulum stress activation via protein kinase R-like endoplasmic reticulum kinase (PERK) phosphorylation after 120 min of incubation [98]. In the case of olanzapine, however, the activation of the downstream mediator eukaryotic translation initiation factor 2α (eIF2α) was attenuated. Olanzapine, but not risperidone, blocked insulin secretion, and both proinsulin and insulin accumulated inside β-cells, explaining in part the accompanying cell death by apoptosis. Of relevance, treatment of isolated rat islets with 50 μM, but not 5 μM, chlorpromazine reduced the protein levels of IRS-2 and protein kinase Cδ after 24 h, without changes to IRS-1 [99]. This decrease was blocked by the 26S proteasomal inhibitors MG-132 or lactacystin, suggesting that chlorpromazine induces IRS-2 degradation via the proteasomal pathway. Therefore, both SGA-induced endoplasmic reticulum stress and insulin signaling impairment in β-cells likely contribute to their dysfunction.

In spite of these data, controversy remains over the mechanism through which SGAs affect insulin secretion. Two scenarios could explain the diabetogenic properties of these drugs based on their effect on β-cells: (1) short-term stimulation of GSIS might lead to β-cell exhaustion in the long-term; (2) short-term inhibition of GSIS might lead to β-cell dysfunction in the long-term. Thus, a better understanding of the molecular mechanisms by which SGAs impact insulin secretion in β-cells and glucagon secretion in α-cells is needed.

### 2.2. Animal Studies Addressing Disruption of Whole-Body Glucose Homeostasis by SGAs: Tissue-Specific Effects in Liver, Skeletal muscle and Pancreas

The metabolic disturbances associated with SGA treatment can be accurately modeled in pre-clinical animal studies, which allow more sophisticated experimental procedures including the analysis of integral tissues and, therefore, provide valuable tools to understand the systemic effects of SGAs that could be translatable to the human population. Of relevance, many studies have used female rodents because of their better suitability for recapitulating AIWG and metabolic side-effects, although with the limitation of the assessment of glucose disturbances derived exclusively from the obesogenic effect of these drugs [100,101]. For this reason, most of the studies reviewed in this section report changes in glucose metabolism attributed to SGAs independently of body weight gain.

#### 2.2.1. SGAs Induce a Metabolic Shift to Fat Oxidation: Insulin-Resistance Condition

In insulin-resistant states, there is a shift in the utilization of carbohydrate fuel to fat fuel, which also explains the typical elevation in plasma glucose levels. In this regard, the use of indirect calorimetry to measure the respiratory exchange ratio (RER), which is used to assess the type of fuel being utilized by the body, has revealed that animals treated with SGAs show a shift from carbohydrate to fat oxidation. For example, acute oral treatment of male C57BL/6 mice with a single dose of olanzapine (2.5 and 5 mg/kg), clozapine (5 and 10 mg/kg) or risperidone (2.5 and 5 mg/kg) induced a dose-dependent lowering of the RER (indicting fat oxidation) within minutes, whereas aripiprazole (1, 5 and 10 mg/kg) had only a modest effect [102]. In the same study, clozapine, risperidone and olanzapine also influenced whole-body cellular respiration by decreasing the rate of oxygen consumption (VO2), but only at the highest doses. The authors reported no changes in RER and VO2 when mice were treated orally with the H1R antagonists astemizole (3 mg/kg) and terfenadine (10 mg/kg), suggesting that H1R is not involved in these responses. Other researchers have shown the same lowering effects of olanzapine for RER and VO2 in male and female wild-type and glucagon receptor-knockout mice treated with a single dose (5 mg/kg, intraperitoneal, i.p.) over 120 min [103], and also in female Sprague-Dawley rats (10 mg/kg, orally) during a 24 h period [104].

#### 2.2.2. The Role of the Liver in Mediating SGAs-Induced Disturbances in Glucose Metabolism

SGA-associated impairment in glucose production, leading to hyperglycemia and hepatic insulin resistance, has been extensively studied in animal models and can be attributed not only to a primary effect in the liver, but also to secondary effects mediated by the CNS. The bulk of studies utilize HEC analysis to assess insulin sensitivity via continuous infusion of insulin. The incorporation of radioactive-labeled glucose can also be used to measure glucose uptake in individual organs. Overall, many studies highlight the liver as a main target for olanzapine and clozapine, the most diabetogenic of the SGAs. In an acute study in male Wistar rats by Houseknecht and collaborators [105] using the HEC technique with continuous infusion of insulin (3 mU/kg/min), subcutaneous (s.c.) administration of clozapine (10 mg/kg) or olanzapine (3.2 and 10 mg/kg) substantially reduced the glucose infusion rate (GIR), an indicator of insulin resistance, and at the same time increased hepatic glucose output (HGO), a measure of both gluconeogenesis and glycogenolysis, but only in those animals receiving the higher dose. Administration of ziprasidone (3.2–30 mg/kg) or risperidone (2 mg/kg) had no effect on these parameters. In line with these results, an increase in HGO and a decrease in GIR was reported in male Sprague-Dawley rats administered olanzapine (3 mg/kg, s.c.) measured by HEC with continuous insulin infusion (5 mU/kg/min) [106], and this was also reported in a similar study in male Sprague-Dawley rats treated with clozapine (10 mg/kg, s.c.) or olanzapine (3 mg/kg, s.c.) [107]. The latter study did not find any impairment in insulin sensitivity with a single-dose of ziprasidone (3 mg/kg, s.c.) or haloperidol (0.25 mg/kg, s.c.). Martins and co-workers [108] compared peripheral and central routes of olanzapine administration in male Sprague-Dawley rats, and found that intravenous (i.v.) olanzapine injection (4.5 mg/kg) or intracerebroventricular (i.c.v.) administration (330 μg) reduced GIR and increased HGO. In a similar study, adult male Wistar rats treated with intragastric olanzapine infusion (30 mg/kg/h) and subjected to either “low” (continuous infusion of insulin at 3 mU/kg/min) or “high” (9 mU/kg/min) HEC analysis showed reduced GIR and hepatic glycogen content, with an increase in HGO detected in the high insulin-infusion group [109]. Another single-dose study described that hepatic glycogen content in fasted female Sprague-Dawley rats was reduced in the first hour after oral administration of clozapine (12 mg/kg), and the rats also developed hyperglycemia [110]. In the setting of repeated dose-experiments, a study in female Sprague-Dawley rats chronically treated with olanzapine (2.0 or 7.5 mg/kg) via osmotic mini-pumps for 4 weeks followed by HEC analysis (insulin infusion at 5 mU/kg/h) revealed reduced GIR and increased HGO in both treatment groups compared with control rats [111]. Overall, irrespective of the different administration routes, doses used and periods of treatment, all of these studies in rat point to an elevation in HGO together with a reduction in GIR—hallmarks of insulin resistance—as well as a reduction of hepatic glycogen content independently of changes in body weight; thus, confirming a direct effect of SGAs on hepatic glucose metabolism.

SGA treatments have been also associated with alterations in the gluconeogenesis pathway in several studies. For instance, upregulation of glucose-6-phosphatase (*G6pc*) mRNA was reported in single-dose experiments of olanzapine (15 nmol, i.c.v.) in male ICR mice [112], in male Sprague-Dawley rats receiving an infusion of olanzapine (prime 22 μg/min for 5 min, then 1.9 μg/min for 60 min, i.c.v.) [108], and in male Wistar rats after an oral chronic treatment with clozapine (10 mg/kg) during 6 weeks [113]. Likewise, an increase in phosphoenolpyruvate carboxykinase (PEPCK) protein levels was reported in the liver of pancreatectomized male Sprague-Dawley rats treated orally with chlorpromazine (50 mg/kg/d) for 8 weeks [114] and also increased mRNA levels were found in the liver of female Sprague-Dawley rats treated for 14 days with clozapine (7.5 mg/kg/d, s.c., twice daily), which was accompanied by augmented mRNA levels of hepatic *Hsd11b1* (encoding 11β-hydroxysteroid dehydrogenase type 1), a key regulatory enzyme of glucocorticoid metabolism linked to T2D [75]. Regarding SGA-induced hepatic metabolic dysfunction, chronic treatment of female C57Bl/6J mice with olanzapine (8 mg/kg/d) via osmotic mini-pumps for 28 days induced anaerobic glycolysis and caused a pseudo-fasted state that depleted hepatic glycogen reserves and activated AMPK signaling. This treatment also increased the hepatic concentration of glutamate and its metabolites, resulting in the activation of mammalian target of rapamycin (mTORC1) signaling in the liver. These data suggest that disturbances in glucose metabolism in the liver caused by olanzapine may be mediated, at least in part, by the simultaneous activation of both catabolic (AMPK) and anabolic (mTORC1) pathways [115]. Importantly, under conditions in which HGO is stimulated by pyruvate administration, a dose-time-dependent hyperglycemic effect of quetiapine was observed in wild-type mice but not in mice with a liver-specific depletion of the circadian clock-component *Bmal1*, pointing to a derangement in the circadian rhythmicity of HGO [116]. Because HGO peaks in the early morning in humans, this study suggests that a daily evening dose of quetiapine might be safer than a morning dose.

Accumulating evidence strongly associates an impairment of hepatic insulin signaling in animals treated with SGAs. Generally, these drugs provoke a decrease in the phosphorylation of AKT, a critical node of insulin signaling in the liver. For example, oral administration of olanzapine (3 mg/kg/d, twice daily) or clozapine (20 mg/kg/d, twice daily) in female Sprague-Dawley rats for 9 weeks led to glucose intolerance and reduced hepatic levels of AKT phosphorylation at Ser^473^ and GSK3β phosphorylation at Ser^9^ levels, in parallel with an up-regulation of H1R and M3R levels [117]. As outlined in the previous sections, most of the diabetogenic properties of SGAs are attributed to their H1R and M3R antagonism. M3R antagonism is related to the inhibition of insulin secretion, but it does not seem to have a role in hepatic glucose homeostasis, as demonstrated in studies in mice lacking M3R in hepatocytes [118]. This latter finding points to the up-regulation of H1R as responsible for the impairment in insulin signaling, with H1R engagement of SGAs inducing weight gain and dysregulation of glucose metabolism by impairing AMPK signaling.

Regarding SGAs-mediated impairment of insulin signaling in the liver, Townsend and collaborators [119] reported that AKT Ser^473^ phosphorylation was completely inhibited in obese male C57BL/6 mice pre-treated for one hour with olanzapine (5 mg/kg i.p.) before an insulin injection, as compared with lean C57BL/6 mice receiving the same treatment. By contrast, Smith and co-workers [78] showed that the effects of daily dosing with clozapine (10 mg/kg, s.c.) during 7 or 28 days did not decrease AKT Ser^473^ phosphorylation. These authors demonstrated that the derangements in glucose metabolism induced by clozapine were mediated by an increase in glucagon secretion, leading to elevated HGO. In a subsequent study, they demonstrated that clozapine simultaneously stimulates insulin and glucagon secretion, a condition that might explain the concurrence of high glucose and high insulin levels in treated animals [92]. Reinforcing these findings, glucagon receptor-knockout male and female C57BL/6J mice were protected against olanzapine (5 mg/kg, i.p.)-induced hyperglycemia in an acute study (120 min), in association with reduced protein content of PEPCK and glucose-6-phosphatase (G6Pase) in the liver [103]. In another study, male Sprague-Dawley rats treated for 8 weeks with olanzapine (5 mg/kg/d, gavage) showed an increase in IRS-1 Ser^307^ and IRS-2 Ser^731^ phosphorylation, which is reported to trigger their proteosomal degradation, resulting in the blockade of insulin signaling [120]. In line with these findings, pancreatectomized male Sprague-Dawley rats treated orally with chlorpromazine (5.50 mg/kg/d) for 8 weeks showed diminished hepatic IRS-2 protein levels and reduced AKT Ser^473^ phosphorylation, together with decreased GIR and increased HGO during HEC analysis [114], all features of hepatic insulin resistance. Interestingly, the authors showed that the metabolic side-effects of chlorpromazine could be mitigated by combining the treatment with exercise. Confirming the impairment of the critical nodes of insulin signaling in the liver by SGAs, an analysis of male Sprague-Dawley rats administered with haloperidol (2 mg/kg/d) or olanzapine (10 mg/kg/d) during 8 weeks via osmotic mini-pumps revealed that only olanzapine decreased hepatic IRS-2 and GSK3α Ser^21^ phosphorylation, and also increased phospho-GSK3β Ser^9^ levels, with haloperidol having no effect [121]. Moreover, the expression of TCF7L2, a key effector of the Wnt signaling pathway strongly associated with glucose homeostasis, was increased in the livers of male C57BL/6 mice treated orally with olanzapine (4 mg/kg/d) for 8 weeks [122]. Reinforcing the local action of SGAs in the liver, antidiabetic drugs that prevent hepatic insulin resistance, such as metformin and rosiglitazone, were found to counteract the metabolic adverse effects of SGAs in a tissue-specific manner [122,123,124].

As alluded to earlier, SGAs can have secondary effects mediated by the CNS and the descending sympathetic and parasympathetic systems, which innervate the liver, skeletal muscle, pancreas, and adipose tissue, and also disrupt glucose homeostasis in these organs. By using central administration routes, several studies have indicated that hepatic insulin resistance can be due to a direct action of these drugs in the orexigenic neurons of the hypothalamus via induction of AMPK, and the subsequent stimulation of hepatic gluconeogenesis through the sympathetic nervous system [108,112]. In this regard, it was previously demonstrated in female Sprague-Dawley rats that oral administration of olanzapine (1 mg/kg, 3 times daily for 8, 16 and 36 days) activates hypothalamic AMPK via blockade of H1R and that this effect is attenuated by the H1R agonist 2-(3-trifluoromethylphenyl) histamine [125]. By contrast, another study [126] found that subchronic oral exposure of female Sprague-Dawley rats to olanzapine (6 mg/kg/day) for 6 days led to the up-regulation of neuropeptide Y and agouti-related protein and the down-regulation of proopiomelanocortin in the arcuate nucleus of the hypothalamus, both in rats fed *ad libitum* and pair-fed. Interestingly, despite weight gain and increased expression of orexigenic neuropeptides, AMPK phosphorylation levels were reduced, an effect not observed after acute administration of either olanzapine or clozapine. Overall, these data suggest that olanzapine-induced hyperphagia is mediated through specific changes in hypothalamic neuropeptides without the requirement of concomitant AMPK activation.

Disruption of M3R density in the brain has also been observed in female SD rats treated with olanzapine (0.25–2.0 mg/kg, self-administered orally in a sweet cookie pellet, 3 times/day for 14 days), which resulted in an up-regulation of M3R in the hypothalamic arcuate ventromedial nuclei and dorsal vagal complex of the brainstem, CNS regions that regulate systemic glucose homeostasis [88]. Elevated hypothalamic M3R levels correlated negatively with peripheral insulin, and positively with ghrelin, cholecystoquinin, food intake and body weight, suggesting that increased M3R density in the CNS is a compensatory mechanism in response to M3R antagonism by olanzapine, which could also disrupt peripheral glucose homeostasis. The promotion of increased HGO secondary to an initial effect in the hypothalamus has also been reported by Girault and co-workers [127], who found that infusion of olanzapine (3 mg/kg) in male RccHan;Han rats activated orexin A-positive neurons in the hypothalamus, which triggered HGO via sympathetic innervation in the liver. Likewise, olanzapine (10 μM) treatment of both male and female C57BL/6J mice reduced the excitability of isolated neurons of the dorsal motor nucleus of the vagus nerve [128], whose activation has been linked to reductions in HGO [129]. Thus, it can be hypothesized that olanzapine-mediated decrease in the vagus nerve neuronal activity would lead to increased HGO. On the other hand, modulation of the endocannabinoid system has been suggested as a good strategy to overcome SGA-induced metabolic side-effects. In a 15-day study in female Wistar rats, olanzapine (4 mg/kg/d, administered by oral gavage twice daily)-activated hypothalamic orexin was attenuated by co-treatment with the cannabinoid receptor type 1 antagonist NESS06SM, (10 mg/kg, administered by oral gavage daily), which also restored pyruvate kinase levels in the liver, strengthening the role of the CNS in modulating peripheral SGAs effects [130].

**Figure 2:**
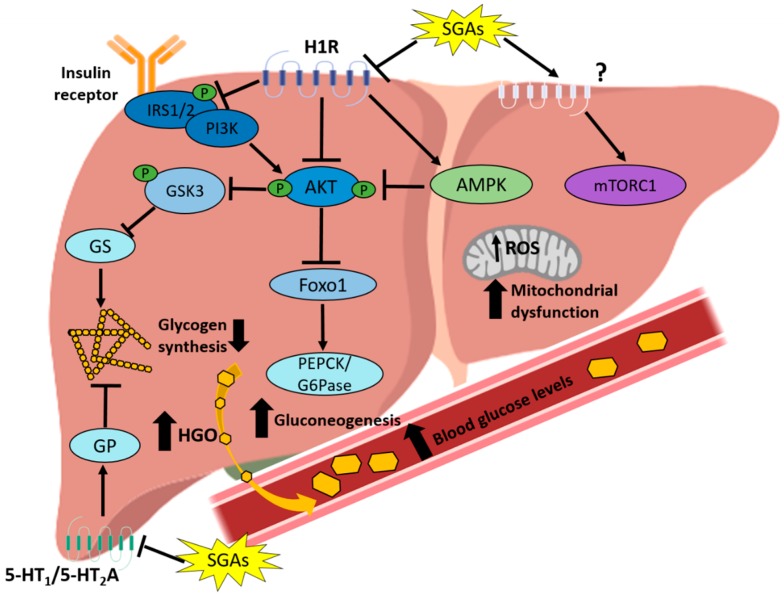
SGA-induced impairment in hepatic insulin signaling and glucose metabolism. SGAs impact the insulin signaling pathway at two levels: inhibition of IRS-1/2 phosphorylation and inhibition of AKT and GSK3 phosphorylation, with H1R antagonism being the proposed SGA target in insulin signaling impairment. Antagonism of 5-HT_1_ and 5-HT_2_A and inhibition of glycogen phosphorylase (GP) would decrease glycogen synthesis, an effect also enhanced by inhibition of glycogen synthase (GS). Both inhibition of glycogen synthesis and stimulation of gluconeogenesis by SGAs increase the hepatic glucose output (HGO), leading to an increase in blood glucose levels. SGAs have been also shown to activate both hepatic catabolic (AMPK) and anabolic (mTORC1) pathways and also induce ROS production and mitochondrial dysfunction.

In contrast to the aforementioned results, some acute SGA studies failed to find CNS-mediated effects on hepatic glucose metabolism. For example, Hahn and co-workers [131] found that a single-dose of olanzapine (75 μg, i.c.v.) in male Sprague-Dawley rats had no effect on HGO and GIR. Furthermore, in the study of Girault and co-workers [109] intragastric administration of olanzapine (3 mg/kg/h) in male Wistar rats led to hyperglycemia and increased corticosterone levels whereas i.c.v. infusion (30 μg/kg/h) failed to change glycemia compared with control rats. However, the lack of effect of olanzapine in this study could be due to the low doses in comparison with, for example, the acute study of Martins and co-workers [108] who reported a central (via i.c.v.) effect of olanzapine with a 330 μg dose. Moreover, a study by Kowalchuk et al. [132] showed that a single-dose of olanzapine (2 mg/kg, s.c.) in male Sprague-Dawley rats during a pancreatic euglycemic clamp, a technique that allows manipulation of central insulin concentrations via i.c.v. infusion of insulin and the concomitant blockade of peripheral insulin action by somatostatin infusion, increased HGO possibly by impairing the ability of central insulin to suppress it. The summarized effects of SGAs on hepatic insulin signaling and glucose metabolism are shown in Figure 2.

#### 2.2.3. Effects of SGAs on Insulin Sensitivity and Glucose Transport in the Skeletal Muscle of Animal Models

As the main tissue involved in insulin-stimulated glucose uptake, insulin resistance and hyperglycemia associated with SGA medication may be generated in the skeletal muscle [109,111]. In agreement with the earlier in vitro studies, a single dose of quetiapine (10 mg/kg, i.p.) downregulated the expression of phosphatidylinositol 3-kinase, regulatory subunit, polypeptide 1 (*Pik3r1*), a critical regulator of the insulin signaling pathway, in the skeletal muscle of male ICR mice fed a chow diet. This effect was reversed in mice fed a dietary cholecalciferol supplement, again suggesting the importance of vitamin D supplementation in SGA-induced metabolic side-effects [77]. Moreover, the beneficial impact of exercise has been tested in female Sprague-Dawley rats treated with olanzapine (10 mg/kg, s.c.) for 9 weeks, with an increase in GLUT4 levels reported in gastrocnemius muscle samples compared with the levels in sedentary rats [133]. While there is no available data on the effects of SGAs on GLUT4 transporter in skeletal muscle, it could be speculated that AMPK activation/expression via H1R blockade could have a direct effect on this tissue by inhibiting translocation, although AMPK activation has been extensively linked to increased glucose uptake in skeletal muscle [134]. A study in male Sprague-Dawley rats examining the effect of olanzapine infusion (1 mg/100 g of body weight for 0.5 h and then 0.04 mg/100 g/h continuously for 23.5 h) on muscle fibers reported an increase in the expression of genes involved in glycolysis, with the exception of the skeletal muscle form of hexokinase (*Hk2*), and some genes of lactate metabolism such as lactate dehydrogenase A (*LdhA*) and the lactate transporter moxocarboxylate transporter 1 (*Mct1*). Olanzapine treatment also resulted in a down-regulation of most of the genes involved in the citric acid cycle, indicating that this drug seems to promote a switch from an oxidative to a more glycolytic fiber type [135].

**Figure 3:**
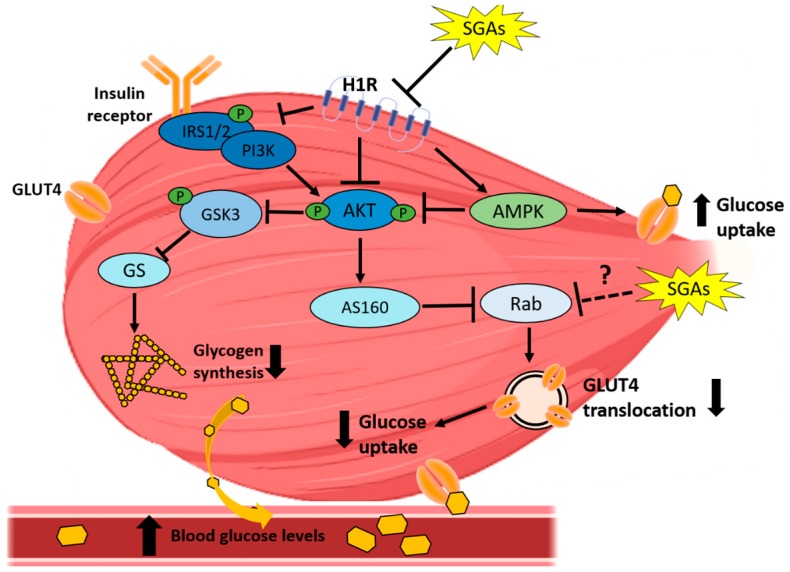
SGA-induced impairment in skeletal muscle insulin signaling and glucose uptake. SGAs impact the insulin signaling pathway at two levels: inhibition of IRS-1/2 phosphorylation and inhibition of AKT and GSK3 phosphorylation, with H1R antagonism being the proposed SGAs target in insulin signaling impairment. SGAs could also increase glucose uptake via AMPK activation, although these drugs most likely exert inhibition of this effect. GLUT4 translocation has been proposed a plausible target for SGAs-reduced glucose uptake in muscle.

In contrast to in vivo data, ex vivo analysis of glucose uptake in isolated epitrochlearis skeletal muscle from male Wistar rats treated with olanzapine (10 mg/kg, s.c.) or clozapine (10 mg/kg, s.c) for 5 days failed to find changes in insulin-stimulated glucose uptake [105]. Similar to the findings in liver, TCF7L2 protein levels were increased in skeletal muscle in male C57BL/6 mice treated with intragastric olanzapine (4 mg/kg/d) for 8 weeks, an effect ameliorated by co-treatment with metformin (150 mg/kg/d) [122]. The summarized effects of SGAs on skeletal muscle insulin signaling and glucose metabolism are shown in Figure 3.

#### 2.2.4. Effects of SGAs on the Endocrine Pancreas: Impairment of Insulin and Glucagon Secretion

As shown by the results in vitro and ex vivo, SGAs directly impact pancreatic islets to regulate insulin and glucagon secretion. Indeed, HEC, which provides an index of the secretory capacity of β-cells, has revealed a direct effect of these drugs on insulin secretion. For instance, in an HEC study in male Sprague-Dawley rats, a single dose of olanzapine (3 mg/kg, s.c.) decreased insulin secretion and C-peptide levels in response to glucose [106]. Similarly, an HEC study assessing the effects of different antipsychotics showed that acute administration of olanzapine (3 mg/kg, s.c.) or clozapine (10 mg/kg, s.c.) to male Sprague-Dawley rats decreased GSIS, whereas risperidone (1 mg/kg, s.c.), haloperidol (0.25 mg/kg, s.c.) and ziprasidone (3 mg/kg, s.c.) had no effect. These findings suggest that the common SGAs olanzapine and clozapine can hinder β-cell compensation through a direct effect on insulin secretion [107]. In repeated-dose studies, female Sprague-Dawley rats treated orally with self-administrated olanzapine (0.25–2 mg/kg, 3 times per day for 14 days) showed reduced fasting insulin levels [136]. Likewise, in a previously-mentioned study, pancreatectomized male Sprague-Dawley rats treated orally with chlorpromazine (50 mg/kg/d) for 8 weeks showed a decrease in the first and second phase of insulin secretion during a hyperglycemic clamp. Interestingly, the islets from these animals showed reduced glucokinase protein levels at the end of the treatment without changes in GLUT2 expression, and this was accompanied by impairment of the insulin signaling pathway as evidenced by a decrease in protein levels of IRS-2, Pdx1 and phosphorylated AKT Ser^473^, similar to the in vitro findings in isolated islets [99]. The same researchers reported these molecular alterations in pancreatectomized and ovariectomized female Sprague-Dawley rats treated orally with olanzapine (2 mg/kg/d) for 8 weeks, also showing decreased β-cell mass. By contrast, risperidone (0.5 mg/kg/d) treatment did not produce significant effects. Interestingly, co-treatment with estrogens reversed the negative impact of olanzapine, suggesting that it should be avoided in the treatment of postmenstrual females [137]. Overall, it could be thought that inhibition of insulin secretion by SGAs is due to blockade of M3R, as previously described in vitro, in addition to impairment of insulin signaling in β-cells. However, in other repeated-dose studies, female Sprague-Dawley rats chronically treated with olanzapine (2.0 or 7.5 mg/kg) with osmotic mini-pumps for 4 weeks showed no changes related to insulin secretion or C-peptide levels compared with animals receiving vehicle [111]. Conversely, continuous infusion of olanzapine (4 or 8 mg/kg/d) for 30 days with mini-pumps in female CD-1 mice led to hyperglycemia in an oral glucose tolerance test and increased serum insulin only at the highest dose used. Interestingly, pancreatic insulin content was elevated in mice treated at the higher dose of olanzapine [138]. Thus, SGAs impair insulin secretion and the insulin-signaling pathway, although the associated molecular mechanisms remain uncertain. Of relevance, the acute inhibitory effect of GSIS by clozapine and olanzapine points to an inhibition of M3R signaling. As we previously mentioned, activation of M3R leads to increased insulin secretion, whereas M3R blockade induces its inhibition [95,139]. The effects are, however, less clear for 5-HT receptors. For example, treatment of male Sprague-Dawley rats with a single dose of MDL100907 (0.5 mg/kg, s.c.), a 5-HT_2_A antagonist, decreases both insulin and C-peptide secretion [140]. Importantly, in human and mouse islets activation of 5-HT_2_B increase GSIS [87]. However, ex vivo studies reported that treatment of islets from *db/db* mice with the 5-HT_2_C antagonist SB242084 (1–10 μM) increases insulin secretion, whereas the 5-HT_2_C agonist m-chlorophenylpiperazine (mCPP) (5–100 μM) inhibits insulin secretion in control islets [141]. In diet-induced insulin resistant states or pregnancy, 5-HT_3_A is necessary for β-cell compensation by increasing insulin secretion [142,143]. Thus, the complex influence of 5-HT signaling, and the different 5-HT receptors targeted by SGAs raises the complexity of the effect of these drugs on insulin secretion. Finally, the increase in insulin secretion mediated by D2R antagonism may predispose to the depletion of insulin granules and β-cell exhaustion in the long term [144]. Of interest, bromocriptine, a D2R agonist, was approved in 2009 as an antidiabetic, suggesting the opposite effect to diabetogenic D2R antagonists [145]. Another study from Hahn and co-workers [131] has suggested that SGAs could impair insulin secretion via centrally mediated mechanisms by addressing the effect of an i.c.v. injection of olanzapine (75 μg) in male Sprague-Dawley rats during a hyperglycemic clamp, reporting decreased insulin and C-peptide secretion.

**Figure 4:**
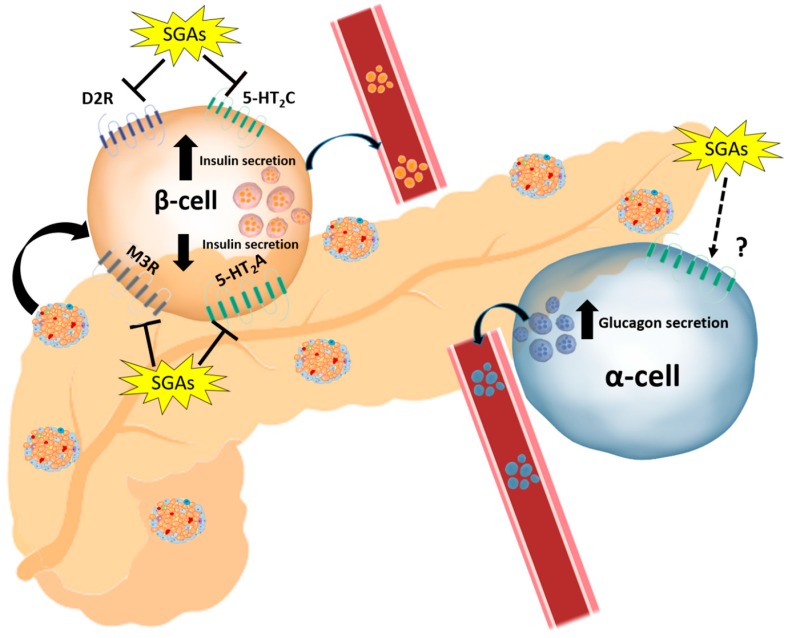
SGA-induced impairment in insulin secretion from β-cells and glucagon secretion from α-cells. The complexity of SGAs impact on insulin secretion is based on the effect mediated by antagonism of SGAs at different receptors. Antagonism of D2R and 5-HT_2_C stimulates insulin secretion, while antagonism of M3R and 5-HT_2_A inhibits this effect. The receptors implicated in the stimulation of glucagon secretion are still unknown.

Beyond the direct and/or indirect effects of APDs in β-cells, several studies report that they increase the levels of glucagon in α-cells [78,92]. Indeed, chronic treatment of male Sprague-Dawley rats with clozapine (10 mg/kg, s.c.) or quetiapine (10 mg/kg, s.c.) for 42 days increased insulin and glucagon levels and decreased GLP-1, an inhibitor of glucagon secretion, in obese and lean rats [146]. This increase in glucagon levels can directly increase HGO, as mentioned previously. In this line, it was reported that the GLP-1 receptor agonist exenatide reversed the damage to pancreatic islets in male C57BL/6 mice treated for 4 months with clozapine (13.5 mg/kg, orally), suggesting a dysregulation of GLP-1-mediated signaling and the subsequent impairment of pancreatic hormone secretion [147]. An increase in glucagon levels after clozapine treatment (5 mg/kg, i.v.) has also been observed in male Wistar rats [148]. The mechanism by which SGAs can stimulate glucagon secretion is, however, not known, but it is unlikely to be due to M3R antagonism, as agonism of these receptors stimulates both insulin and glucagon secretion, as demonstrated in mice with genetic deletion of this receptor [139]. The increase in glucagon secretion and subsequent stimulation of HGO can also stimulate insulin secretion, which could explain the hyperinsulinemia found in patients despite the inhibitory effect of SGAs on GSIS in the initial stages of the treatment. It is worth mentioning that the hyperinsulinemia associated with these drugs can directly impact on AKT/GSK3 signaling in other tissues. For instance, activation of the AKT/GSK3 pathway in the brain of fasted male Sprague-Dawley rats after clozapine treatment (10 mg/kg) was reported to be due to an increase in the levels of insulin (which can cross the blood-brain barrier) and not to a direct effect of the drug [149]. Thus, although current evidence points to SGA-induced glucagon release, the effects on α-cells are not known and more research is needed. The summarized effects of SGAs on beta-cells insulin secretion and alpha-cells glucagon secretion are shown in Figure 4.

### 2.3. Completing the Preclinical Model Picture: Genetic and Epigenetic Determinants of SGAs-Induced Metabolic Side-Effects in Humans

Studies in animal models have been instrumental in identifying the molecular mechanisms underlying the metabolic side-effects of SGAs with techniques that cannot be applied to humans. Nevertheless, minimally invasive procedures in schizophrenia and SGA-treated patients have helped to unravel the potential genetic component of the variability of metabolic side-effects among patients. To date, most of the studies investigating the potential genetic drivers of SGA-induced metabolic side-effects have used highly accessible tissues such as blood leukocytes or peripheral blood mononuclear cells, although some studies have used extracted DNA from tissue biopsies. There are many excellent reviews addressing the genetic and epigenetic markers of both schizophrenia *per se* and the differential response to SGAs [150] as well as AIWG [151,152], and so here we will only illustrate the genetic traits of the main molecular effectors in SGA-induced dysregulation of glucose homeostasis.

Several genes expressed in skeletal muscle have emerged as potential candidates for SGA-induced metabolic dysfunction. Given the strong influence of these medications on *AKT* expression levels, gene-specific methylation patterns in the promoters of *AKT1*, *2* and *3* were analyzed in fasting skeletal muscle biopsies from 16 patients with bipolar disorders treated with an SGA (quetiapine, risperidone, olanzapine, asenapine or aripiprazole) and compared with 14 patients treated with mood stabilizers for at least 3 months. Methylation of *AKT1* and *AKT2* was higher in SGA-treated patients than in patients treated with mood stabilizers, whereas no differences were found in *AKT3* methylation [153]. Accordingly, increased *AKT* promoter methylation might explain the decreased insulin sensitivity in skeletal muscle associated with treatment, as increased methylation in the gene promoters is generally linked to decreased gene expression and functionality. In a similar study, Emamian et al. [154] screened various protein kinases and phosphatases in 28 individuals with schizophrenia and 28 controls, finding significant differences only for AKT1 protein levels. They also compared post-mortem brains of patients with schizophrenia and control individuals, and also found a decrease in AKT1 protein and phosphorylated GSK3β in the hippocampus and frontal cortex of individuals with schizophrenia, pointing to AKT1 as a possible susceptibility marker for schizophrenia. Other mediators of the insulin-signaling pathway have not been associated with SGA-induced metabolic side-effects. In a cross-sectional study of 438 patients, Moons and colleagues failed to identify an association between DNA methylation of *IGF1* and *IGF2* promoters and SGA-induced metabolic side-effects [155]. In a study searching for candidate genes in skeletal muscle from 195 patients with schizophrenia medicated with SGAs, a moderate association was found between the polymorphism rs9852 variant in *TBC1* domain family member 1 protein (*TBC1D1*), a Rab-GTPase that regulates GLUT4 trafficking, and lowers weight gain, although the functional relevance is unknown [156]. Interestingly, a previous study demonstrated that TBC1D1 is a substrate of AKT and AMPK and that *Tbc1d1*-deficient mice have impaired insulin-stimulated glucose transport and reduction of GLUT4 abundance in skeletal muscle [157]. Thus, gene polymorphisms in *TBC1D1* could explain disturbances in glucose metabolism with SGAs and different responses between patients.

Other potential candidate genes have been discovered by focusing on gene variants that can affect β- and α-cell functionality. For example, the rs10423928 polymorphism (A/T) in the β-cell glucose-dependent insulinotropic polypeptide receptor (*GIPR*), which stimulates insulin secretion upon binding of the incretin GIP, was associated with increased insulin levels in an oral glucose tolerance test in patients with schizophrenia treated with olanzapine [158]. As discussed earlier, long-term treatment with high-risk diabetogenic SGAs has been also linked to decreased GLP-1 levels in rats [146], and the positive effects of GLP-1 analogs have been proved in animals [147] and humans [159,160]. Correspondingly, *GCG* (encoding for a preprotein cleaved into four different proteins, including GLP-1 and glucagon) and *GLP1R* have been assessed as potential pharmacogenetic markers of antipsychotic response. In an analysis of 216 patients with schizophrenia treated with clozapine or olanzapine, Brandl and collaborators [161] found an association between rs13429709, a single-nucleotide polymorphism located near GCG, and weight gain. In a similar analysis, the Clinical Trial of Antipsychotic Intervention Effectiveness (CATIE) study examined whether genetic variation in *GLP1R* impacted the response to different SGAs, finding that different patient haplotypes had different responses to SGAs, but were not associated with AIWG [162]. A better understanding of the physiological impact of these GLP1R variants might highlight insulin and glucose disturbances associated with the treatments. The CATIE trial also evaluated 106 single-nucleotide polymorphisms in synaptic vesicle protein 2 (*SV2C*), also implicated in glucose-dependent insulin release, finding that four of them correlated with olanzapine and quetiapine response [163]. Furthermore, haplotypes in the serotonin receptor *HTR2A* [−1438A, −783A, 102T, and 452Tyr] had a protective effect against the clozapine and olanzapine-mediated increase in C-peptide levels [164].

In another study analyzing DNA methylation changes in candidate genes, Burghardt and colleagues [165] used epigenome-wide association studies to identify a hypomethylated CpG site in fatty acyl Coa reductase 2 (*FAR2*) associated with insulin resistance as measured by homeostatic model assessment. Also, single-nucleotide polymorphisms in the melacortin 4 receptor (*MC4R*), a common biomarker for predicting individual susceptibility to weight gain and obesity, have been linked to SGA-induced MetS [166]. Another potential genetic determinant is the 5-HT_2_C receptor (*HT2RC*), which has been previously associated with feeding behavior and weight gain and which is antagonized by clozapine and olanzapine. Mulder and collaborators [167] reported an association between several polymorphisms at *HT2RC* (HTR2C:c.1–142948(GT)n, rs518147 (−697 G/C), and rs1414334) and an increased risk for MetS in patients treated with olanzapine, risperidone or clozapine. An association between MetS and *HT2RC* has also been proposed in other studies. For example, the rs498177 variant showed a significant association with MetS in female patients treated with olanzapine or risperidone [168], and the same was found for the rs518147CC and rs12836771GG variants in patients [169]. Although the contribution of *HT2RC* to MetS can be because of AIWG, we cannot rule-out the dysregulation of glucose metabolism. A recent study comparing the gene expression patterns in blood samples between first-episode, drug-naïve patients with schizophrenia with weight gain after 3 months of treatment with risperidone or aripiprazole with those of patients that did not gain weight reported an enrichment of genes involved in immune system pathways in the former, highlighting the role of inflammation in the metabolic disturbances associated with SGAs [170]. Moreover, a study addressing the clinical response to clozapine found genetic variability in two genes involved in the hypothalamic-pituitary-adrenal axis: *FKBP5* (encoding for FK506 binding protein 5) and *NTRK2* (encoding for neurotrophic tyrosine kinase receptor 2). It is possible that this genetic variability might be responsible for the key role of the CNS in mediating the metabolic side-effects of SGAs [171]. Finally, it is noteworthy to mention that, in the last years, microRNAs (miRNAs), which have emerged as important post-transcriptional regulators of gene expression, have been involved in the occurrence of neurological disorders including schizophrenia [172]. Among them, miRNA-9 and miRNA-326 are regulators of human *DRD2* expression in human dopaminergic neurons [173]. Of relevance, a recent study has revealed that the combination of miRNA-22-3p, miRNA-92a-3p, and miRNA-137, detected in peripheral blood, was closely associated with schizophrenia [174]. A step further, the decrease of miRNA-21 expression found in patients treated with olanzapine, quetiapine, ziprasidone or risperidone [175], points to possible changes in circulating miRNAs expression in response to antipsychotic medication for schizophrenia although the molecular mechanisms beyond of this need further research.

## 3. Concluding Remarks

Antipsychotics, especially SGAs, are associated with weight gain and glucose dysregulation, two major contributors to T2D development. While AIWG is associated with glucose dysregulation, evidence points to a direct effect of SGAs on glucose metabolism. In this review, we have addressed how diabetogenic SGAs, in particular olanzapine and clozapine, but also the FGA chlorpromazine, can impair insulin signaling in insulin-sensitive tissues, such as liver and skeletal muscle, interfering with glucose transport, glycogen synthesis and gluconeogenesis and, consequently, inducing insulin resistance (Table 1). Importantly, SGAs have a direct effect on β-cell function and insulin secretion and probably also on α-cells and associated glucagon secretion (Table 1). Overall, the development of insulin resistance and the impact on β-cell function associated with antipsychotics, especially SGAs, can explain the diabetogenic effects associated with treatment. Moreover, the existence of different polymorphisms in the human population likely explains the different susceptibility of patients with schizophrenia to the metabolic side-effects of SGAs.

## Figures and Tables

**Table 1:** List of mentioned APDs effects on peripheral insulin-sensitive tissues inducing dysregulation in glucose homeostasis.

Pharmacological Target	Antipsychotics Mentioned in this Review	Effect in Insulin-Sensitive Tissue
**H1R antagonism**	Olanzapine, clozapine	Hepatic insulin signaling impairment [73,114,117,119,120,121,122]
	Olanzapine	Hepatic AMPK signaling impairment [115,124]
	Olanzapine	Skeletal muscle AMPK signaling impairment [74]
	Olanzapine, clozapine, quetiapine	Skeletal muscle insulin signaling impairment [77,135]
**5-HT_1_ agonism**	Olanzapine	↓ Hepatic glycogen synthesis [67]
**5-HT_2_A antagonism**	Olanzapine	↓ Hepatic glycogen synthesis [67,105,110]
	Quetiapine	↓ Skeletal muscle glucose uptake [77]
**D2R antagonism**	Haloperidol, sulpiride, olanzapine, clozapine	↑ Glucose-Stimulated Insulin Secretion [85,92]
	Clozapine	↑ Basal insulin secretion [90,91]
	Chlorpromazine	Hepatic insulin signaling impairment [100]
**M3R antagonism**	Clozapine, olanzapine	↓ Insulin secretion [95,96]
**Unknown**	Clozapine, olanzapine, quetiapine	↑ Glucagon secretion [92,146,148]
	Clozapine, quetiapine	↓ GLP-1 levels [146]
	Olanzapine	↓ Skeletal muscle glycogen synthesis [71]
		↓ Skeletal muscle glucose uptake [72]
